# How does green credit guidelines affect environmentally friendly enterprises’ ESG? A quasi-natural experiment from China

**DOI:** 10.1371/journal.pone.0304384

**Published:** 2024-05-29

**Authors:** Shengyu Xu, Jinqiu Yang, Ruile Li

**Affiliations:** School of Economics and Management, Chongqing Jiaotong University, Chongqing, China; Inner Mongolia University, CHINA

## Abstract

Following decades of extensive economic development, promoting the transition to greening and decarbonization in economic development have become inevitable choices for controlling environmental pollution and achieving high-quality development in China. Green Credit Guidelines (NIGCG) is a major policy innovation to promote green credit and further improve sustainable economic development. The influence of these guidelines on environmentally friendly enterprises’ sustainable development capacity, proxied by environmental, social, and corporate governance (ESG), has not yet been discussed. Therefore, this study takes the NIGCG issued in 2012 as a quasi-natural experiment, and adopts a propensity score matching–difference-in-differences (PSM-DID) model to test whether the NIGCG has affected ESG in environmentally friendly enterprises from 2009 to 2022. Our results indicate that the NIGCG significantly boosts environmentally friendly enterprises’ ESG, and this finding remains robust to a series of tests. In addition, a mediating effect analysis reveals that the NIGCG affects enterprises’ ESG through research and development (R&D) investment, verifying the Porter hypothesis in China. Finally, we determine that the role of NIGCG in promoting ESG is significantly reflected in the non-politically connected enterprises and enterprises in the eastern region. The empirical results suggest that the authorities should stimulate enterprises’ R&D investments through supporting policies, such as tax reimbursement and government subsidies, and formulate differentiated policies according to the characteristics of enterprises and their regions, so as to improve the effect of NIGCG.

## 1. Introduction

Since the Twelfth Five-Year Plan, China has witnessed enormous and rapid economic development. However, this extensive economic development mode caused increasingly serious problems such as environmental pollution, resource depletion, as well as ecological damage. The Chinese authorities have begun to attach importance to environmental protection as well as transitioning the traditional development mode to a sustainable development strategy in recent years. The development of green credit and provision of more social investments for enterprises’ upgrading and transformation is the key to advancing enterprises sustainable development. Under these circumstances, China issued the Green Credit Guidelines (NIGCG) on February 24, 2012, aiming to achieve sustainable development by establishing a green credit guide for financial institutions and increasing support for the green, low-carbon and circular economy. Studying the relationship between the NIGCG and environmental, social, and corporate governance (ESG) in environmentally friendly enterprises is conducive to an in-depth understanding the policy effect of NIGCG and provides policy references for realizing sustainable economic development.

As a critical indicator, ESG is commonly applied to evaluate enterprises’ sustainable development capacity. Therefore, scholars have dedicated considerable research to explore the potential determinants of ESG by examining the performance of enterprise (Lins et al., 2017; Giuli and Kostovetsky, 2014) [1, 2], green finance (Xu et al., 2023; Zhang, 2023a; Xue et al., 2023) [3–5], corporate leadership (Dabbebi et al., 2022; Burke, 2021; Liu and Zhang, 2023; Ritz, 2022) [6–9], ownership (Rees and Rodionova, 2015; Nofsinger et al., 2019; Weber, 2014) [10–12], and environmental regulation (Chen et al., 2022; Yan et al., 2022; He et al., 2023; Shu and Tan, 2023; Lu and Cheng, 2023) [13–17]. Nonetheless, there is a lack of discussion remains regarding the relationship between the NIGCG and environmentally friendly enterprises’ ESG.

Regarding the influence of environmental and green financial policies, there are two controversial viewpoints: a) Porter hypothesis (Porter and Van der Linde, 1995), suggesting that suitable environmental regulations can stimulate enterprises’ innovation activities, enhance production efficiency as well as competitiveness [18]; b) the restriction hypothesis (Jaffe et al., 1995), arguing that stringent environmental regulations can increase the environmental costs of enterprises, crowding out research and development (R&D) investments, and harm productivity [19]. Thus, it is meaningful to understand which of the above two hypotheses are dominated in the NIGCG’s effect on environmentally friendly enterprises’ ESG.

To fill this research gap, we use the data of Chinese A-shared listed enterprises during 2009–2022, and employ a propensity score matching–difference-in-differences (PSM-DID) approach to investigate the relationship between the NIGCG and enterprises’ ESG. Since the primary purpose of NIGCG is to strategically develop green credit by promoting banks’ green focus and has a significant influence on improving social comprehensive, coordinated, and sustainable development, this policy may contribute to environmentally friendly enterprises’ ESG. Therefore, this study classifies sample enterprises into environmentally friendly enterprises (treatment group) as well as other enterprises (control group). We conduct a common trend test to determine whether PSM-DID is suitable for this research. Moreover, we expect a certain lag in the policy effect. Our benchmark regression confirms that the NIGCG has significantly improved environmentally friendly enterprises’ ESG, and this finding remains robust to a series of tests.

In addition, the mediating effect analysis demonstrates that the investment of R&D is the intermediary channel for NIGCG to affect enterprises’ ESG, verifying the Porter hypothesis for China. This study also employs two heterogeneity tests to discuss the different effects of NIGCG on enterprises’ ESG, including politically connected (PC) enterprises and non-PC enterprises as well as enterprises located in different regions. The empirical findings indicate that the promoting effect of NIGCG on ESG is mainly reflected in enterprises that the non-politically connected and enterprises in the eastern region.

The theoretical contributions of this study include the following. a) Previous research has studied the relationship between the NIGCG and heavy polluters; however, this policy predominantly aims to promote green industrial development. Therefore, our study explores the influence of the NIGCG on environmentally friendly enterprises’ sustainable development capacity at the micro level, which enriches the research related to the effect of policy’s micro level. b) The mediating effect analysis confirms that the NIGCG can motivate enterprises to increase the investment of R&D and enhance production efficiency, and ultimately boosts enterprises’ ESG, verifying the Porter hypothesis in China.

Moreover, the practical contributions are as follows. a) The mediating effect analysis reveals how the NIGCG affects environmentally friendly enterprises’ ESG, suggesting that the authorities should stimulate enterprises’ R&D investments through supporting policies, such as tax reimbursement and government subsidies. b) Because the NIGCG has different effects on various enterprise types, the government should appropriately adjust this policy to emphasize strategic support for specific enterprises. c) The findings of our research may also help other emerging markets attempting to implement green finance policies that are similar to the NIGCG for promoting sustainable economic development.

The rest of this study is organized as follows: In Section 2, we review related literature and develop hypotheses; In Section 3, we introduce methodology, including data and sample, variables, and summary statistics; In Section 4, we report results of empirical analyses; and we conclude this study in Section 5.

## 2. Literature review and research hypotheses

### 2.1 Literature review

To examine how environmental as well as environmental finance policies affect the economy, scholars have conducted a number of research. However, the effect of such policies remains controversial.

Some scholars support Porter hypothesis (Porter and Van der Linde, 1995), which asserts that suitable environmental regulations can motivate enterprises to engage in innovation activities that enhance production efficiency as well as competitiveness [18], including Jaffe and Palmer (1997) [20], Ramanathan et al. (2017) [21], Xue et al. (2023) [5], Wang (2023) [22], Yu et al. (2023) [23], Yan et al. (2022) [14], Zhang et al. (2021) [24], Zhang (2023b) [25], Xu et al. (2023) [3], Li et al. (2022b) [26], Chi and Yang (2023) [27], He et al. (2024) [28], Zhang and He (2024) [29] and Wang et al. (2023) [30]. For instance, Jaffe and Palmer (1997) found environmental compliance expenditure to be positively related to enterprises’ R&D spending [20]. Ramanathan et al. (2017) determined that enterprises’ private benefits from sustainability activities are generally better obtained through actively focusing on environmental regulations and environmental performance [21]. Zhang et al. (2021) and Xue et al. (2023) found that local the returns on high ESG portfolios green financial policies significantly increase corporate ESG performance [5, 24]. Wang (2023) also showed that green finance policies are positively associated with environmentally friendly industries’ green innovation efficiency [22]. Yan et al. (2022) noted that green financial reform significantly reduces enterprises’ agency costs as well as increases the scale of R&D investment, boosting investment efficiency [14]. Furthermore, green financial pilot policies can also significantly improve enterprises’ financing scale, and boost sustainable development capacity (Xu et al., 2023; Yu et al., 2023; Zhang, 2023b) [3, 23, 25]. Li et al. (2022b) demonstrated that green finance reduces the enterprise debt cost by improving the social responsibility of enterprises [26]. Chi and Yang (2023) claimed that green policies can help achieve green economic transformation using market-oriented governance [27]. He et al. (2024) and Zhang and He (2024) found that green financial system can promote ESG and total factor productivity of environmentally friendly enterprises [28, 29]. Wang et al. (2023) found that low-carbon pilot cities can boost urban carbon emission efficiency by elevating the standard of urban innovation and advanced urban industrial structure [30].

In contrast, some other scholars support the effect of compliance cost, arguing that stringent environmental regulations can increase the environmental costs of enterprises and harm productivity, including Gray (1987) [31], Gollop and Roberts (1983) [32], Testa et al. (2011) [33], Zhao et al. (2023) [34], Tang et al. (2020) [35] as well as Hou et al. (2020) [36]. For example, Gollop and Roberts (1983) showed that emissions regulation limitations would cause a notable increase in enterprises’ production costs [32]. Gray (1987) noted that environmental regulation weakens the enterprises’ productivity growth [31]. Testa et al. (2011) argued that direct regulation can significantly harm enterprises’ competitiveness in terms of innovation, intangibility, and green business performance [33]. Hou et al. (2020) demonstrated that it is difficult to advance green total factor productivity while improving the environment vias the sulfur dioxide emissions trading scheme [36]. Tang et al. (2020) found that command-and-control environmental regulation is negatively related to enterprises’ total factor productivity [35]. Zhao et al. (2023) found that the green finance reform affect enterprises’ total factor productivity not through technological innovation, but through environmental protection investments as well as financing constraints [34].

Finally, some studies have indicated an uncertain relationship between environmental finance policies, environmental policies, and the economy. For example, Alpay et al. (2002) found that U.S. contamination regulations cannot affect the food manufacturing industry’s profitability or productivity in U.S. [37]. Becker (2011) demonstrated that counties with higher environmental compliance costs have no statistically significant impact on the average manufacturing plant’s productivity [38]. Wang and Shen (2016) studied the impact of environmental regulation on China’s environmental productivity and found that the effect of environmental regulation on productivity is different in industries [39]. Wang et al. (2018) showed that water quality regulation does not significantly affect surviving enterprises’ productivity [40].

### 2.2 Research hypotheses

The China Banking Regulatory Commission formulated NIGCG on February 24, 2012 to adjust the credit structure, prevent environmental and social risks, better serve the real economy, and further promote the transformation of economic development mode and economic restructuring. Banking financial institutions should improve green credit, increase support for green economy, low-carbon economy, and circular economy, prevent the threat of uncertain environment and society, and further promote the transformation and diversification of quality levels and development models.

Our review of previous research indicates that the impacts of these environmental as well as environmental finance policies on the economy remains controversial. Notably, although a few scholars have explored the relationship between the NIGCG and the ESG of enterprises (Li et al., 2022a; Gao and Liu, 2023; Lei et al., 2023), these studies primarily examined whether the NIGCG affected heavy polluters’ ESG [41–43]. As noted above, the NIGCG aims to promote green credit development by banking institutions to advance sustainable development; therefore, we contend that the NIGCG will also have an impact on environmentally friendly enterprises’ ESG. Furthermore, this study demonstrates that environmental finance policies can increase enterprises’ investment on R&D, which contributes to enterprises’ sustainable development capacity. Therefore, we assert that the NIGCG may affect environmentally friendly enterprises’ R&D investment, which improves such enterprises’ ESG. To verify our conjecture, this study proposes the following hypothesis:

**H1:** NIGCG implementation affects environmentally friendly enterprises’ ESG.
**H2:** NIGCG implementation affects environmentally friendly enterprises’ ESG through R&D investment.

## 3. Data and methodology

### 3.1 Sample description

This study examines all A-share listed enterprises on Chinese stock markets from 2009 to 2022 as our initial sample. We choose this sample period because data for several variables, such as ESG, are unavailable prior to 2009, and the relevant data are only available until 2022. Finally, the relevant enterprise data are obtained from the CSMAR Database, and the data of ESG are derived from the Sino-Securities’ ESG rating index.

We further clean sample enterprises based on the following six criteria. (1) The ST, *ST and PT listed enterprises are removed as our research focuses on general cases rather. (2) Enterprises in financial sector are deleted, because of the differences in regulation. (3) To control for the influence of the life cycle effect on enterprise operation, the listed enterprises in the Growth Enterprise Market as well as in the Science and Technology Innovation Board are excluded. (Enterprises listed on the Growth Enterprises Market and the Science and Technology Innovation Board Market are smaller than those enterprises listed on the main board. Moreover, the former are normally in the growth stage, while the latter are in the maturation stage.) (4) Enterprises with missing values are excluded from the analysis. (5) We also exclude enterprises with only a first enterprise-year observation because of the IPO effect on enterprises. (6) Finally, to control for extreme values, we winsorize our sample at a 1% level. Totally, the sample period from 2009 to 2022 includes 2,989 enterprises.

### 3.2 Variables definition

#### 3.2.1 Dependent variable

As mentioned above, ESG can reflect the sustainable development capacity of enterprises. Our study uses Sino-Securities’ ESG data as the dependent variable. Sino-Securities’ ESG data mainly consists of two forms: a) a rating index, that offers AAA, AA… C ratings for listed enterprises, and b) an overall score, that provides numerical scores for listed enterprises. We adopt both of these data as our dependent variables. We transform the ratings index into numbers for our data analysis. For instance, AAA to 9, AA equals to 8, etc., where a larger number indicates an enterprise’s stronger sustainable development capacity.

#### 3.2.2 Independent variables

We employ *EFTim*e as the core independent variable, which assigned a value of 1 if the observation is post-event and belongs to an environmentally friendly enterprise, otherwise 0. As noted previously, the NIGCG was implemented on February 24, 2012; thus, we define 2009–2011 as pre-event and 2012–2022 as post-event. We classify sample enterprises into environmentally friendly enterprises (treatment group) as well as others (control group). This study screen environmentally friendly enterprises as follows: a) Environmentally friendly enterprises listed on Peking University’s China Center for Economic Research, the CSMAR, and RESSET databases, and b) environmentally friendly enterprises based on Environmental Industry Climate Index Report. This study identified 157 environmentally friendly enterprises and 2,832 others in our sample.

**Fig 1:**
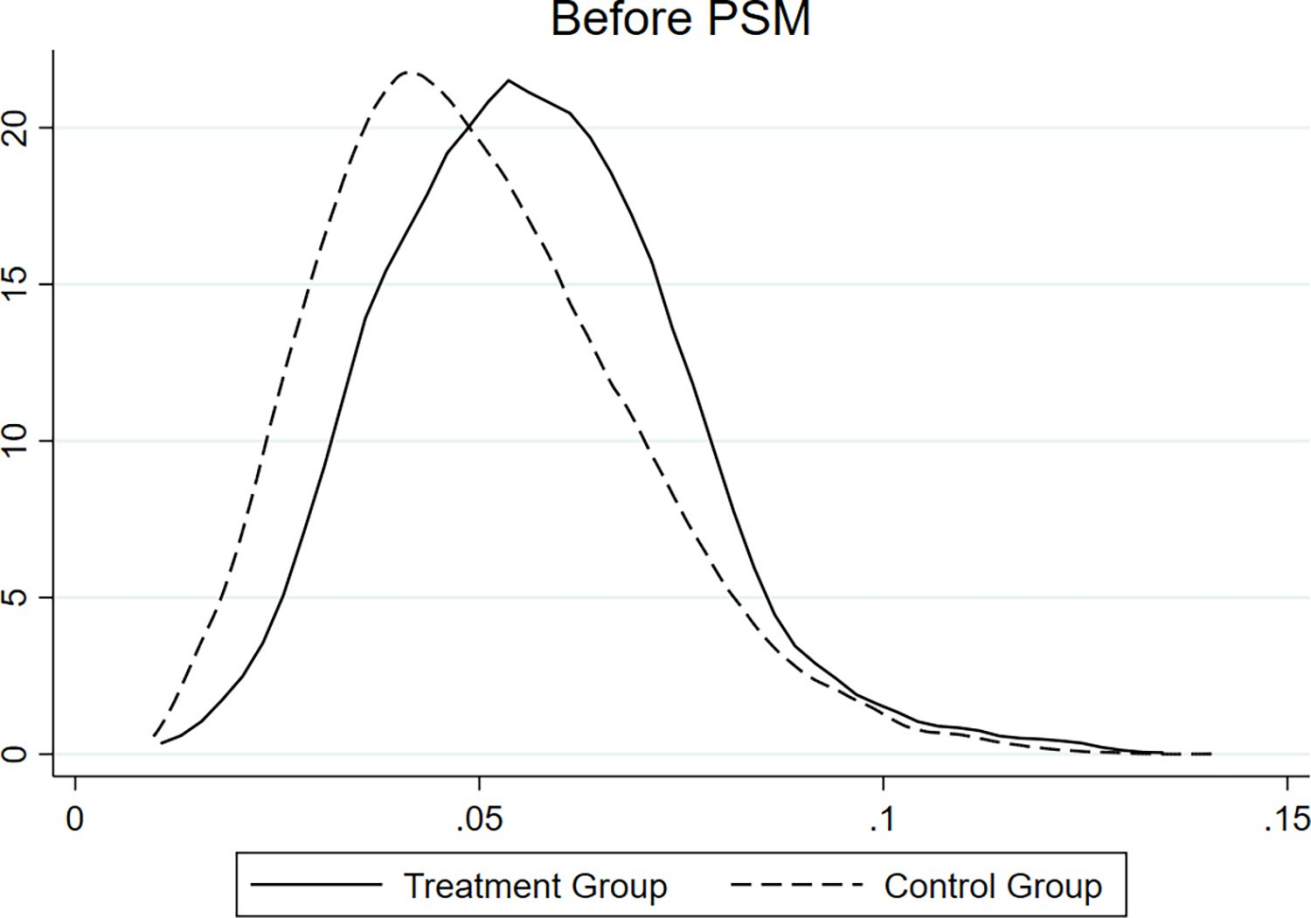
Before PSM. This figure shows the kernel density curve before PSM.

**Fig 2:**
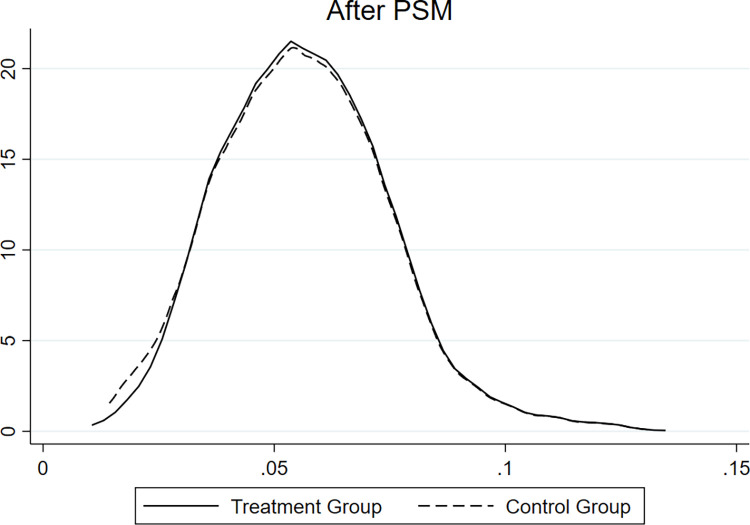
After PSM. This figure shows the kernel density curve after PSM.

Fig 1 presents the considerable differences between the treatment and control groups. To eliminate the endogeneity issue caused by potential sample selectivity bias, our research adopts the PSM-DID model to examine whether and how the NIGCG affected environmentally friendly enterprises’ ESG during the sample period. For PSM, we use logit regression for empirical analysis with the matching caliper set to 0.05 and employ a nearest-neighbor, 1:1, no-release matching. Finally, we obtain 157 environmentally friendly enterprises (1,486 enterprise-year observations) and 668 other enterprises (1,486 enterprise-year observations). Fig 2 reveals that the differences across the treatment and control groups become significantly smaller after PSM screening.

According to the research of Rees and Rodionova (2015), Weber (2014), Burke (2021) and He et al. (2023) [7, 10, 12, 15], the enterprise-level attributes may also have significant effects on ESG. Therefore, we select the corporate governance and financial fundamental as control variables. Corporate governance consists of three variables, *Board size*, *First*, as well as *Sedtenth*. Financial fundamentals also contain three variables, *Age*, *Lev*, and *Cash Flow*. Appendix A reports the detail of variable definitions.

### 3.3 Descriptive statistics

The descriptive statistics corresponding to the study’s variables are presented in Table 1. It can be found that the mean of *ESG* is 4.1800, and its maximum and minimum values are 7.0000 and 1.0000. The mean of *ESG Score* is 73.3925, and its maximum and minimum values are 85.2600 and 55.270. *EFTime*’s mean value is 0.4277, which indicate that 42.77% of enterprises are categorized as the post-event treatment group. In addition, the mean of *First*, *Sedtenth*, *Board Size*, *Age*, *Lev*, and *Cash Flow* are 0.3787, 0.2232, 2.2342, 2.3456, 0.3639, and 0.2555, respectively. For the definitions of all variables, see Table 2.

**Table 1:** Descriptive statistics.

Variables	Obs	Mean	Median	Std. Dev.	Max	Min
ESG	2,972	4.1800	4.0000	1.0680	7.0000	1.0000
ESG Score	2,972	73.3925	73.5550	5.1663	85.2600	55.2700
EFTime	2,972	0.4277	0.0000	0.4948	1.0000	0.0000
First	2,972	0.3787	0.3686	0.1563	0.7889	0.0728
Sedtenth	2,972	0.2232	0.2112	0.1258	0.5758	0.0174
Board Size	2,972	2.2342	2.3026	0.1831	2.7726	1.7918
Age	2,972	2.3456	2.4849	0.7206	3.4340	1.0986
Lev	2,972	0.3639	0.3186	0.2192	0.9597	0.0510
Cash Flow	2,972	0.2555	0.2199	0.1730	0.6222	0.0057

This table presents the results of descriptive statistics. For variable definitions, please refers to Table 2.

**Table 2:** Variable definitions.

Variable	Definition
ESG	The Sino-Securities’ ESG data in rating, which is a widely used enterprise-level sustainable development capacity indicator.
ESG Score	The Sino-Securities’ ESG data in score, which is a widely used enterprise-level sustainable development capacity indicator.
EFTime	Dummy variable, equals to one if the observation is an environmentally friendly enterprise and is post-event, and zero otherwise.
EF2009	Dummy variable, equals to one if the observation is an environmentally friendly enterprise in 2009, and zero otherwise.
EFTime1	Dummy variable, equals to one if the observation is an environmentally friendly enterprise and is post-2009, and zero otherwise.
EFTime2	Dummy variable, equals to one if the observation is an environmentally friendly enterprise and is post-2010, and zero otherwise.
First	Shareholding ratio of the largest shareholder.
Sedtenth	Shareholding ratio of the second to tenth largest shareholders.
Board size	The natural logarithm of the number of directors plus one.
Age	The natural logarithm of listed years plus one.
Lev	The ratio of total liabilities to total assets.
Cash Flow	The ratio of cash and cash equivalents to total assets.
R&D	The ratio of research and development investments to total assets.

This table presents the variable definitions.

Table 3 demonstrates that the correlation coefficient between variables is small, excepting *ESG* and *ESG Score*. The correlation coefficient between *ESG* and *ESG Score* is 0.9623; however, these two factors are dependent variables and would not be used in the same regression model. Thus, the multicollinearity issue is unlikely to be a problem in our empirical analysis.

**Table 3:** Correlation matrix.

	ESG	ESG Score	EFTime	First	Sedtenth	Board Size	Age	Lev
ESG Score	0.9623***							
	(0.000)							
EFTime	-0.0795***	-0.0787***						
	(0.000)	(0.000)						
First	0.1263***	0.1327***	-0.2220***					
	(0.000)	(0.000)	(0.000)					
Sedtenth	0.0488***	0.0482***	0.0729***	-0.3925***				
	(0.008)	(0.009)	(0.000)	(0.000)				
Board Size	-0.0462**	-0.0449**	0.1462***	-0.0519***	-0.0085			
	(0.012)	(0.014)	(0.000)	(0.005)	(0.642)			
Age	-0.1473***	-0.1558***	0.2307***	-0.2268***	-0.3021***	0.0882***		
	(0.000)	(0.000)	(0.000)	(0.000)	(0.000)	(0.000)		
Lev	-0.1044***	-0.1093***	0.6332***	-0.1806***	-0.0508***	0.1687***	0.3370***	
	(0.000)	(0.000)	(0.000)	(0.000)	(0.006)	(0.000)	(0.000)	
Cash Flow	0.1526***	0.1564***	-0.6743***	0.1363***	0.1026***	-0.1081***	-0.2781***	-0.6509***
	(0.000)	(0.000)	(0.000)	(0.000)	(0.000)	(0.000)	(0.000)	(0.000)

Note: This table presents the results of correlation matrix. P-values are in parentheses. ***, ** and * refer to the significance at the 1%, 5% and 10% levels, respectively. (Hereinafter inclusive)

## 4. Empirical analyses

### 4.1 Common trend test

The essential precondition of applying PSM-DID model for policy effect analysis is that the environmentally friendly enterprises and others should satisfy the common trend assumption. Reference to Beck et al. (2010) [44], we establish Eq (1) to examine whether there is a common trend before and after NIGCG implementation between environmentally friendly and other enterprises as follows:

$$Y_{i,t}=\alpha_{i,t}+\beta_{i,t}^{1}{EF2009}_{i,t}+\beta_{i,t}^{2}{EF2010}_{i,t}+\ldots +\beta_{i,t}^{7}{EF2022}_{i,t}+\theta_{i,t}^{1}{Governance}_{i,t}+\theta_{i,t}^{2}{Fundamentals}_{i,t}+\varepsilon_{i,t}$$ (1)

where *ESG* and *ESG Score* represent the dependent variables; *EF2009* stands for a dummy variable, which assigned to 1 if the observation is an environmentally friendly enterprises in 2009 and 0 otherwise; *Governance* consists of *Board size*, *First*, and *Sedtenth*; *Fundamentals* include *Age*, *Cash Flow*, *and Lev*; and *i* refers to enterprise i and *t* is time t.

**Fig 3:**
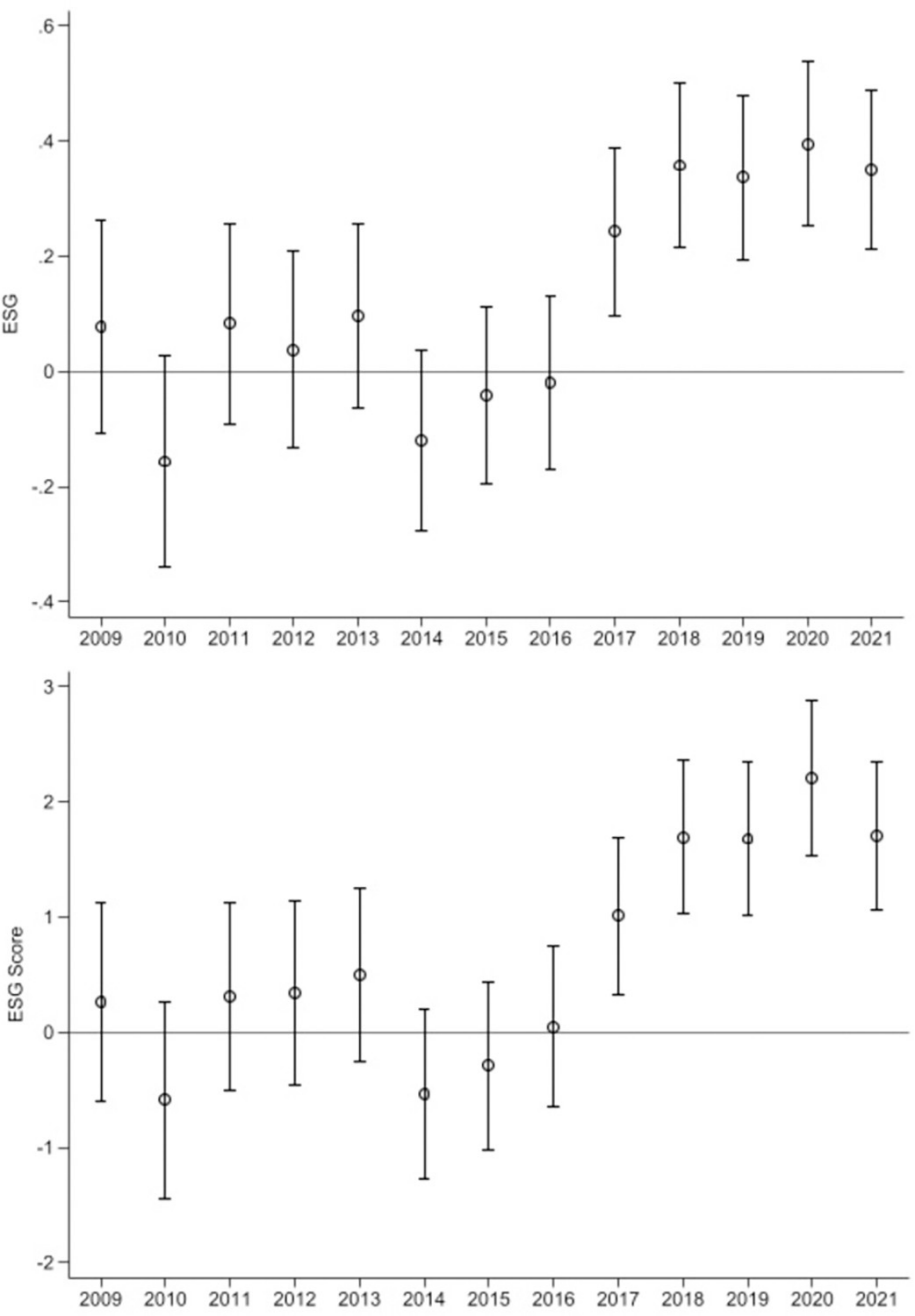
Common trend test. This figure shows the common trends of ESG and ESG Score, respectively.

As shown in Table 4, the coefficients’ confidence intervals prior to NIGCG implementation (2009–2011) are all 0, whereas the coefficients after NIGCG implementation gradually deviate, and the confidence intervals do not include 0 from 2017 forward. These results satisfy the common trend assumption (although a long lag effect is apparent for the NIGCG affecting enterprises’ ESG); therefore, the PSM-DID approach is appropriate for our study. A possible reason for the relatively long lag in the policy effect may be that although NIGCG implementation encourages the provision of financing services for environmentally friendly enterprises, it takes a long time for environmentally friendly enterprises to upgrade production process after receiving funds to improve ESG level. Finally, we present Fig 3 based on the regression results to directly reflect the common trend test’s results.

**Table 4:** Dynamic effect analysis.

	ESG	ESG Score
EF2009	0.0775	0.2576
	(0.489)	(0.623)
EF2010	-0.1561	-0.5898
	(0.159)	(0.255)
EF2011	0.0825	0.3058
	(0.435)	(0.536)
EF2012	0.0380	0.3393
	(0.714)	(0.483)
EF2013	0.0962	0.4932
	(0.324)	(0.280)
EF2014	-0.1202	-0.5400
	(0.208)	(0.226)
EF2015	-0.0416	-0.2920
	(0.659)	(0.507)
EF2016	-0.0196	0.0461
	(0.829)	(0.913)
EF2017	0.2427***	1.0115**
	(0.006)	(0.014)
EF2018	0.3581***	1.6950***
	(0.000)	(0.000)
EF2019	0.3366***	1.6810***
	(0.000)	(0.000)
EF2020	0.3956***	2.2056***
	(0.000)	(0.000)
EF2021	0.3498***	1.7060***
	(0.000)	(0.000)
First	0.7693***	3.5627***
	(0.000)	(0.000)
Sedtenth	0.4176**	1.7376*
	(0.044)	(0.077)
Board Size	-0.1485	-0.8977
	(0.249)	(0.143)
Age	-0.0698*	-0.4073**
	(0.076)	(0.031)
Lev	-0.4012***	-2.3290***
	(0.006)	(0.001)
Cash Flow	0.4840***	2.1573***
	(0.004)	(0.007)
Random Effect	Yes	Yes
Observations	2,972	2,972
R-squared	0.0567	0.0597

This table reports the results of dynamic effect analysis.

### 4.2 Benchmark regression

We test whether the NIGCG can improve environmentally friendly enterprises’ sustainable development capacity through Eq (2).

$$Y_{i,t}=\alpha_{i,t}+\beta_{i,t}{EFTime}_{i,t}+\theta_{i,t}^{1}{Governance}_{i,t}+\theta_{i,t}^{2}{Fundamentals}_{i,t}+\varepsilon_{i,t}$$ (2)

where *EFTime* presents the dummy variable, which assigned to 1 if the observation is post-event and belongs to an environmentally friendly enterprise, otherwise 0. The other variables are shown in Eq (1).

As reported in Table 5, the coefficient of *EFTime* with *ESG* (*ESG Score*) is 0.1116 (0.6048) and significant at 5%, which means that the NIGCG can significantly boost environmentally friendly enterprises’ ESG (Score). This finding strongly supports Hypothesis 1. Moreover, we find that *First*, *Sedtenth*, and *Cash Flow* (*Lev*) are positively (negatively) related to environmentally friendly enterprises’ sustainable development capacity (*ESG* and *ESG Score*). The results are similar when we apply ESG and ESG Score as independent variables.

**Table 5:** Benchmark analysis.

	ESG	ESG Score
EFTime	0.1116**	0.6048**
	(0.037)	(0.017)
First	0.8358***	3.9057***
	(0.000)	(0.000)
Sedtenth	0.6315***	2.7987***
	(0.002)	(0.004)
Board Size	-0.1755	-1.0231*
	(0.172)	(0.095)
Age	-0.0272	-0.1837
	(0.461)	(0.299)
Lev	-0.3189**	-1.9344***
	(0.028)	(0.005)
Cash Flow	0.4589***	2.0840***
	(0.007)	(0.009)
Random Effect	Yes	Yes
Observations	2.972	2,972
R-squared	0.0422	0.0445

This table presents the results of our benchmark analysis.

### 4.3 Counterfactual test

We also conduct counterfactual tests (policy implementation time and replacing the control group) to verify the empirical results robustness. First, we establish Eq (3) for policy implementation time as follows:

$$Y_{i,t}=\alpha_{i,t}+\beta_{i,t}{EFTime1/EFTime2}_{i,t}+\theta_{i,t}^{1}{Governance}_{i,t}+\theta_{i,t}^{2}{Fundamentals}_{i,t}+\varepsilon_{i,t}$$ (3)

This research assumes that the establishment periods of NIGCG were in 2010 and 2011, respectively, changing the sample period to 2009–2011. Therefore, the term, *EFTime1* (*EFTime2*), assigned to 1 if an observation is environmentally friendly and is in 2010–2011 (2011) and 0 otherwise. Remaining variables are described in Eq (1). As shown in Table 6, it can be found that *EFTime1* and *EFTime2* become insignificant, verifying that the empirical results are robust.

**Table 6:** Policy implementation time.

	ESG	ESG Score	ESG	ESG Score
EFTime1/ EFTime2	-0.0668	-0.1884	0.1227	0.4932
	(0.455)	(0.642)	(0.181)	(0.232)
First	0.5933	2.8819*	0.5873	2.8517
	(0.105)	(0.098)	(0.107)	(0.101)
Sedtenth	0.3997	-0.0708	0.3000	-0.4500
	(0.426)	(0.976)	(0.549)	(0.850)
Board Size	-0.0436	0.1744	-0.0641	0.1021
	(0.869)	(0.890)	(0.807)	(0.935)
Age	-0.0064	-0.2105	-0.0226	-0.2795
	(0.948)	(0.653)	(0.818)	(0.550)
Lev	0.3351	1.1315	0.2620	0.8772
	(0.276)	(0.437)	(0.388)	(0.543)
Cash Flow	0.8301**	4.1234**	0.9342**	4.4485***
	(0.024)	(0.018)	(0.010)	(0.009)
Random Effect	Yes	Yes	Yes	Yes
Observations	486	486	486	486
R-squared	0.0407	0.0438	0.0429	0.0457

This table presents the results of policy implementation time.

Furthermore, we also exclude environmentally friendly enterprises in the sample and randomly select half of the enterprises (334 enterprises) from others as the treatment group. Table 7 reveals that NIGCG implementation has an insignificant effect on enterprises’ ESG after reexamining the NIGCG’s influence with the new control group, indicating that this policy only affects the treatment group, which validates the robustness of the baseline empirical results.

**Table 7:** Replacing control group.

	ESG	ESG Score
EFTime	0.0028	0.1136
	(0.962)	(0.683)
First	0.6102**	2.4500**
	(0.012)	(0.036)
Sedtenth	0.2698	0.6025
	(0.359)	(0.665)
Board Size	0.0542	0.2848
	(0.749)	(0.723)
Age	-0.1096**	-0.6567***
	(0.022)	(0.004)
Lev	0.3033	1.4742
	(0.221)	(0.209)
Cash Flow	0.3227	1.2815
	(0.120)	(0.189)
Random Effect	Yes	Yes
Observations	1,486	1,486
R-squared	0.0360	0.0379

This table presents the results of replacing control group.

### 4.4 Additional tests

#### 4.4.1 Mediating effect analysis

We have demonstrated that NIGCG implementation can significantly boost environmentally friendly enterprises’ ESG, which preliminarily verifies the Porter hypothesis in China. This study next adopts R&D as a mediating variable to examine the role of R&D in the relationship between the NIGCG and environmentally friendly enterprises’ ESG. The equations are established as follows:

$$Y_{i,t}=\alpha_{i,t}+\beta_{i,t}^{1}{EFTime}_{i,t}+\theta_{i,t}^{1}{Governance}_{i,t}+\theta_{i,t}^{2}{Fundamentals}_{i,t}+\varepsilon_{i,t}$$ (4)

$${R\&D}_{i,t}=\alpha_{i,t}+\beta_{i,t}^{2}{EFTime}_{i,t}+\theta_{i,t}^{1}{Governance}_{i,t}+\theta_{i,t}^{2}{Fundamentals}_{i,t}+\varepsilon_{i,t}$$ (5)

$$Y_{i,t}=\alpha_{i,t}+\beta_{i,t}^{3}{EFTime}_{i,t}+\beta_{i,t}^{4}{R\&D}_{i,t}+\theta_{i,t}^{1}{Governance}_{i,t}+\theta_{i,t}^{2}{Fundamentals}_{i,t}+\varepsilon_{i,t}$$ (6)

where *R&D* is the mediating variable, which is the proportion of R&D investments to total assets. We use R&D to measure enterprises’ scientific and technological innovation capacity. Other variables are presented in Eq (1).

From columns (1)-(6) of Table 8, we confirm that *EFTime* is positively related to ESG and R&D. Moreover, columns (3) and (6) of Table 8 indicate that R&D is positively related to ESG. A mediating effect account for 9.93% and 9.26%, respectively. This finding demonstrates that the NIGCG can affect sample enterprises’ ESG through R&D investment, Verifying the Porter hypothesis in China, which supports Hypothesis 2. Furthermore, Wu et al. (2023) discovered that enterprises concentrating on the digital economy are inclined to augment their R&D [45].

**Table 8:** Mediating effect model.

Column	(1)	(2)		(3)	(4)	(5)		(6)
	ESG	R&D		ESG	ESG Score	R&D		ESG Score
EFTime	0.1367** (0.017)	0.0031*** (0.002)		0.1231** (0.031)	0.7653*** (0.006)	0.0031*** (0.002)		0.6944** (0.012)

R&D				4.4351***				23.1622***
				(0.000)				(0.000)
First	0.8473***	-0.0228***		0.9484*** (0.000)	4.2415***	-0.0228***		4.7698*** (0.000)
	(0.000)	(0.000)			(0.000)	(0.000)		
Sedtenth	0.4611**	-0.0042		0.4796**	2.1193**	-0.0042		2.2157**
	(0.014)	(0.199)		(0.010)	(0.019)	(0.199)		(0.014)
Board Size	-0.1583	-0.0041**		-0.1401	-0.7054	-0.0041**		-0.6107
	(0.137)	(0.026)		(0.187)	(0.169)	(0.026)		(0.233)
Age	-0.1121***	-0.0048***		-0.0910***	-0.5978***	-0.0048***		-0.4876***
	(0.000)	(0.000)		(0.004)	(0.000)	(0.000)		(0.002)
Lev	0.0642	-0.0215***		0.1596	0.1942	-0.0215***		0.6923
	(0.610)	(0.000)		(0.211)	(0.749)	(0.000)		(0.261)
Cash Flow	0.9719***	0.0076***		0.9382***	4.8532***	0.0076***		4.6771***
	(0.000)	(0.008)		(0.000)	(0.000)	(0.008)		(0.000)
Sobel test			0.0136 (z = 2.486; p = 0.013)				0.0709 (z = 2.552; p = 0.011)	
Bootstrap test (Direct effect)			0.1231 (z = 2.270; p = 0.023)				0.6944 (z = 2.890; p = 0.004)	
Bootstrap test (Indirect effect)			0.0136 (z = 2.740; p = 0.006)				0.0709 (z = 2.990; p = 0.003)	
Proportion of indirect effect			9.93%				9.26%	
Adj-R^2^	0.0461	0.1231		0.0514	0.0502	0.1231		0.0564
F statistics	21.50	60.57		21.12	23.41	60.57		23.20
Obs	2,972	2,972		2,972	2,972	2,972		2,972

This table reports the results of mediating effect analyses.

#### 4.4.2 Heterogeneity tests

We employ two heterogeneity tests to examine whether NIGCG implementation has various effects on PC enterprises and those located in different regions. In this study, we reference Chen et al. (2011), Francis et al. (2009), and Wu et al. (2012) [46–48] and use the chairman or CEO as a current or former government official to quantify PC, employing Eq (2) for regression analysis. Table 9 reveals that *EFTime* has an insignificant influence on PC enterprises’ sustainable development capacity, while it has a significant effect on those without connections. In addition, the impacts of *First* and *Sedtenth* on politically connected enterprises’ sustainable development capacity are larger than those of non-PC enterprises. *Board Size* and *Age* have increased negative effects on non-PC enterprises, while the impacts on PC enterprises are insignificant.

**Table 9:** Subsample test—PC vs. non-PC.

	Panel A: PC		Panel B: Non-PC	
	ESG	ESG Score	ESG	ESG Score
EFTime	-0.0031	0.0475	0.1620**	0.8221**
	(0.972)	(0.911)	(0.018)	(0.011)
First	1.1099***	4.9135***	0.6513***	3.3095***
	(0.000)	(0.001)	(0.004)	(0.002)
Sedtenth	0.7813**	3.5680**	0.4788*	2.0835*
	(0.030)	(0.039)	(0.063)	(0.088)
Board Size	0.2814	1.4059	-0.3025*	-1.5931**
	(0.220)	(0.205)	(0.054)	(0.033)
Age	0.0964	0.4154	-0.1104**	-0.5346**
	(0.134)	(0.186)	(0.015)	(0.014)
Lev	-0.2568	-2.0558*	-0.2786	-1.5766*
	(0.308)	(0.093)	(0.115)	(0.059)
Cash Flow	0.5723*	1.8628	0.4691**	2.4684**
	(0.050)	(0.184)	(0.024)	(0.012)
Random Effect	Yes	Yes	Yes	Yes
Observations	942	942	2,030	2,030
R-squared	0.0409	0.0323	0.0500	0.0556

This table reports the results of additional test—subsample test (PC).

Furthermore, we separate our enterprises into three regional sub-samples, including eastern, middle, and western China and use Eq (2) for regression analysis. The empirical results reported in Table 10 reveals that NIGCG implementation has effectively improved enterprises’ ESG in the eastern region, while this promotional impact is not evident in the middle as well as western regions. *First* has a significant effect in all three regions, *Sedtenth* has a relatively large role in promoting ESG in the eastern and middle regions. *Board Size* only affects the western region, *Lev* only affects the middle region, and *Cash Flow* only affects the eastern region.

**Table 10:** Subsample test—eastern vs. middle vs. western.

	Panel A: Eastern		Panel B: Middle		Panel C: Western	
	ESG	ESG Score	ESG	ESG Score	ESG	ESG Score
EFTime	0.1664**	0.8163***	-0.1817	-0.5770	0.1183	0.6431
	(0.011)	(0.008)	(0.174)	(0.371)	(0.368)	(0.301)
First	0.6603***	2.8566***	1.0383**	5.9597***	1.5417***	6.7128***
	(0.003)	(0.007)	(0.014)	(0.004)	(0.002)	(0.005)
Sedtenth	0.5207**	2.0377*	1.1352**	6.0055**	0.4591	1.9840
	(0.037)	(0.084)	(0.027)	(0.015)	(0.371)	(0.421)
Board Size	-0.1516	-0.7914	0.1732	0.6112	-0.5073*	-2.9891**
	(0.336)	(0.291)	(0.616)	(0.717)	(0.077)	(0.030)
Age	-0.0613	-0.3230	0.1506	0.5349	0.0241	-0.0782
	(0.163)	(0.123)	(0.129)	(0.272)	(0.806)	(0.869)
Lev	-0.2296	-1.3489	-0.6360*	-3.8185**	-0.4484	-2.6752
	(0.192)	(0.106)	(0.079)	(0.030)	(0.220)	(0.124)
Cash Flow	0.4263**	1.9496**	0.6268	2.3683	0.7384	3.7070*
	(0.035)	(0.041)	(0.141)	(0.255)	(0.104)	(0.087)
Random Effect	Yes	Yes	Yes	Yes	Yes	Yes
Observations	2,125	2.125	436	436	411	411
R-squared	0.0290	0.0286	0.1570	0.1660	0.1148	0.1219

This table reports the results of additional test—subsample test (region). Eastern region contains Liaoning, Beijing, Tianjin, Hebei, Shandong, Jiangsu, Shanghai, Zhejiang, Fujian, Guangdong, Hainan and Guangxi; Middle region contains Heilongjiang, Jilin, Inner Mongolia, Shanxi, Henan, Anhui, Hubei, Jiangxi and Hunan; Western region contains Xinjiang, Tibet, Gansu, Qinghai, Sichuan, Yunnan, Guizhou, Chongqing, Shaanxi and Ningxia. Hong Kong, Macau and Taiwan are excluded from our sample due to data unavailability.

## 5. Conclusions and recommendations

China’s government has recognized that the urgency of environmental protection while continuing to promote high economic growth to advance sustainable development. Under these circumstances, China issued the NIGCG on February 24, 2012, aiming to achieve sustainable social development goals by developing green credit by banking financial institutions and increasing support for the green, low-carbon, and circular economy. Considering the importance and comprehensiveness of this policy, this research constructs a quasi-natural experiment and adopts the PSM-DID model to empirically explore the impact of NIGCG on environmentally friendly enterprises’ ESG during 2009–2022.

The conclusions are as follow: The NIGCG significantly boosts environmentally friendly enterprises’ sustainable development capacity, which is measured by ESG and ESG Score, although a certain lag effect is noted. This finding holds after conducting several robustness tests. Additionally, the mediating effect analysis reveals that the NIGCG can affect enterprises’ ESG through R&D investment, verifying the Porter hypothesis in China. Furthermore, results of heterogeneity tests show that the role of NIGCG in improving enterprises’ ESG is significantly reflected in the non-PC enterprises and enterprises in eastern region.

Based on the empirical results, we suggest the authorities to offer some supporting policies, such as tax reimbursement and government subsidies, to enterprises, so as to stimulate their R&D investments and promote the NIGCG’s effect. Besides, differentiated policies should also be formulated according to the characteristics of enterprises and their regions, which could enhance the NIGCG’s promotional effect nationwide.

## Supporting information

S1 Data (ZIP)
